# Potential Value of Circular RNA circTBC1D4 in Gastrointestinal Stromal Tumors

**DOI:** 10.1155/2022/9019097

**Published:** 2022-05-24

**Authors:** Chunyan-Zou, Yanpeng-He, Changshun-Xie, Fulai-Gao, Xiaoli-Xie, Xiukun-Chai, Chenxi-He, Dongqiang-Zhao

**Affiliations:** ^1^Department of Gastroenterology, The Second Hospital of Hebei Medical University, Shijiazhuang, Hebei, China; ^2^Department of Gastroenterology, The First Hospital of Qinhuangdao, Qinhuangdao, Hebei, China; ^3^Department of Gastroenterology, Affiliated Xing Tai People Hospital of Hebei Medical University, Xingtai, Hebei, China

## Abstract

**Aims:**

To explore the expression of circular RNA (circRNA) in gastrointestinal stromal tumors.

**Background:**

Gastrointestinal stromal tumors (GIST) are mainly distributed in the stomach and small intestine. Recently, it has been verified that circular RNA (circRNA) has an important function in the regulation of GIST. Nevertheless, detailed investigations of circRNA-miRNA-mRNA regulatory networks in GIST are lacking.

**Objective:**

To analyze the gastrointestinal stromal tumor circRNA-miRNA-mRNA network, assessing the effect of circle RNA in gastrointestinal stromal tumors.

**Method:**

All the differential circRNAs and mRNAs were obtained from Gene Expression Omnibus (GEO) microarray data (GSE131481 and GSE147303, GSE131481, and GSE13861). Furthermore, a circRNA-miRNA-mRNA network was established. Gene Ontology (GO) analysis and Kyoto Encyclopedia of Genes and Genomes (KEGG) enrichment were used to reveal the correlation between the functions of signaling pathways and target genes. The hub genes of protein-protein interaction (PPI) network and cytoHubba were also defined. Quantitative real-time PCR (qRT-PCR) was used to measure the expression levels of hsa-circ-0002917 (circTBC1D4), hsa-miR-590-5p (miR-590-5p), and PLN.

**Results:**

PPI network and Cytoscape showed that ATP1A2, PLN, KCNMA1, and SCNN1B were four central DEGs. GO analysis results revealed that DEGs were involved in negative management of myocardial contraction, regulation of myocardial cell contraction, ethanol oxidation, cellular potassium ion homeostasis, and relaxation of cardiac muscle, and KEGG analysis showed that major DEGs were with cGMP-PKG signaling pathway. Moreover, we obtained two pairs of axes, namely, hsa-circ-0039216/hsa-miR-338-3p/ATP1A2 and hsa-circ-0002917/hsa-miR-590-5p/PLN. The target of TBC1D4 is miR-590-5p, and miR-590-5p increased after knocking down TBC1D4. Moreover, PLN was the target of miR-590-5p, and miR-590-5p exerts antitumor effects by reducing PLN.

**Conclusions:**

In this study, we constructed a circRNA-miRNA-mRNA management network interrelated with GIST and researched the potential roles of circRNA. Moreover, we discovered a new molecular landmarker for the prediction, diagnosis, and therapy of patients.

## 1. Introduction

A gastrointestinal stromal tumor (gastrointestinal stromal tumor (GIST)) is the general common gastrointestinal tumor with an overall incidence of 0.68/100,000 [1]. GIST is mainly distributed in the stomach (55.6%) and small intestine (31.8%) [2]with phenotypes ranging from benign to malignant [3]. Several main tumor biomarkers are applied to the diagnosis and prediction of GIST. In particular, the development of circulating biomarkers still has a significant impact on the prognosis of gastrointestinal stromal tumors. Abnormal molecules with changing progress may be used to explore reliable biomarkers, which in turn could improve the management of GIST [4]. Moreover, investigating the underlying mechanisms of the occurrence and development of GIST could lead to the achievement of early diagnosis, effective treatment, and good prognosis of GIST.

circRNA is an endogenous noncoding RNA with a covalently closed loop structure whose 3′ and 5′ ends are noncollinearly connected by a procedure of “reverse splicing” [5]. The poverty of a 5′ cap and 3′ tail makes circRNA more stable against exonuclease than linear RNA [6]. circRNAs are widely expressed mainly as miRNA sponges in different species, thus alleviating miRNA target inhibition [7]. They also competitively impede the binding of miRNA and the mRNA targets [8]. The biological function of a great majority of circRNAs is still unclear. Some researchers found that circ-PRKCI was significantly upregulated in esophageal cancer[9]. Li et al. shown that circSMARCA5 had a diagnostic value for HCC[10]. Lu et al. found that the tumor volume in the circ-FBXW7 overexpression group was significantly lower in colorectal cancer[11]. Therefore, we created a circRNA-miRNA-mRNA network to move forward a single step to assess the role of circRNA and mRNA dysregulation in GIST.

In this research, we found 7 differentially expressed circRNAs (DEcircRNAs) and 12 differentially expressed genes (DEGs) by analyzing two groups of circRNAs and mRNA expression profiles in the GEO dataset. We also performed GO and KEGG analysis on key DEGs to research the role of DEGs. Protein-protein interaction (PPI) network was created, and four key DEGs and one eventful module of the network were defined. The Circular RNA Interactome and TargetScanHuman databases were used to predict miRNAs, and the circRNA-miRNA-mRNA network was succeed in building, which was used to further explore the role of circTBC1D4/hsa-miR-590-5p/PLN axes in stromal tumors.

## 2. Materials and Methods

### 2.1. Microarray Data Source

The data applied in the report were retrieved from the GEO database (https://www.ncbi.nlm.nih.gov/geo/). Using the keyword “gastrointestinal stromal tumor” to search on the GEO, two circRNA arrays were obtained from GSE131481 and GSE147303 databases; the platforms were GPL22120 and GPL21825. At the same time, two mRNA expression sequences were obtained from GSE131481 and GSE13861 databases; the platforms were GPL22120 and GPL6884. Both circRNA databases included 3 GIST tissues and 3 normal gastric tissues. At the same time, the mRNA databases of GSE131481 included 3 GIST tissues and 3 normal tissues, and GSE13861 included 3 GIST tissues and 19 normal tissues. Because the data in the GEO database publicly available, no ethical approval or informed consent was required for this study.

### 2.2. Differential Expression Analysis

The original data were normalized and performed using log_2_ transformation. The Bioconductor Limma package was useful for definitude of differentially expressing circRNAs (DEcircRNAs) and mRNAs in each dataset [12]. The edge R software package was used for screening criterion for ∣log2fold change (FC) | >1 and *p*value < 0.05 for the differential expression circRNAs (DEcircRNAs); the Venny 2.1.0 (https://bioinfogp.cnb.csic.es/tools/venny/) was used for constructing a Venn diagram to obtain differentially expressed DEcircRNAs [13]. The mRNA dataset of GSE13861 (DEmRNAs) screening criterion for ∣log2FC | >2 and adjusted *p* value < 0.05; as the |log2FC| of most DEmRNAs in GSE131481 was less than 2, we set the criterion ∣log2FC | >1 and adjusted *p* value of <0.05 to be defined as statistically obvious; the intersections of DEmRNAs were DEGs.

### 2.3. GO Functional Analysis and KEGG Pathway

In order to evaluate the main functional pathways of DEGs, DAVID (https://david.ncifcrf.gov/) was applied to annotate DEGs for GO, and ggplot2 software package in R studio and R scripting language was used to analyze the KEGG pathway. The *p* value < 0.05 was considered the criterion.

### 2.4. PPI Network and Hub Genes

On the basis of the identifying DEGs, the PPI network was created by using the Interaction Gene Retrieval (STRING) database, and required confidence (combined score) ≥ 0.4 was considered the cut-off criterion for PPI extraction. Visualization was presented using Cytoscape 3.6.1. The cytoHubba plug-in was applied to reveal the critical DEGs by a node level. The MCODE application was used for screening hub gene modules from the PPI network, where cut-off ≥ 2, node score cut-off = 0.2,*k*‐score ≥ 2, and*Max*.depth = 100were cut-off criteria [14].

### 2.5. miRNA Binding Sites

The Circular RNA Interactome (https://circinteractome.irp.nia.nih.gov/) was applied to predict miRNA-binding sites, where CSCD (https://gb.whu.edu.cn/CSCD/) showed the binding sites (MRE) of circRNAs. The TargetScanHuman (http://www.targetscan.org/) was used to forecast the interaction between mRNA and miRNA. Intersecting miRNAs in the two databases were thought in target miRNAs.

### 2.6. circRNA-miRNA-mRNA Network

The circRNA-miRNA-mRNA network was framed by combining circRNA-miRNA and miRNA-mRNA. Finally, it was visible using Cytoscape 3.6.1.

### 2.7. Quantitative Real-Time PCR (qRT-PCR)

The cell line was GIST-882.After isolating total RNA from cells (Solarbio, Beijing, China),quantitative real-time PCR (qRT-PCR)was applied to find the expression of circTBC1D4, miR-590-5p, and PLN (Hifair®). The primers in our study were as follows: 5′-GCCACCCACCTTCAAGCACAA-3′ (F) and 5′-CAGAGTCAGCATTACCTCATCAACCT-3′ (R) for hsa-circ-0002917, 5′-TCAGACTTCCTGTCCTGCTGGTATC-3′ (F) and 5′-GCAGAACTTCAGAGA AGCATCACGAT-3′ (R) for PLN, and 5′-GTGAAGGTCGGTGTGAACGGATT-3′ (F) and 5′-CGTGAGTGGAGTCATACTGGAACAT-3′ (R) for GAPDH. Relative expression quantification was compared using the 2-*ΔΔ*CT method.

## 3. Results

### 3.1. DEcircRNAs and DEGs in Gastrointestinal Stromal Tumors

**Figure 1:**
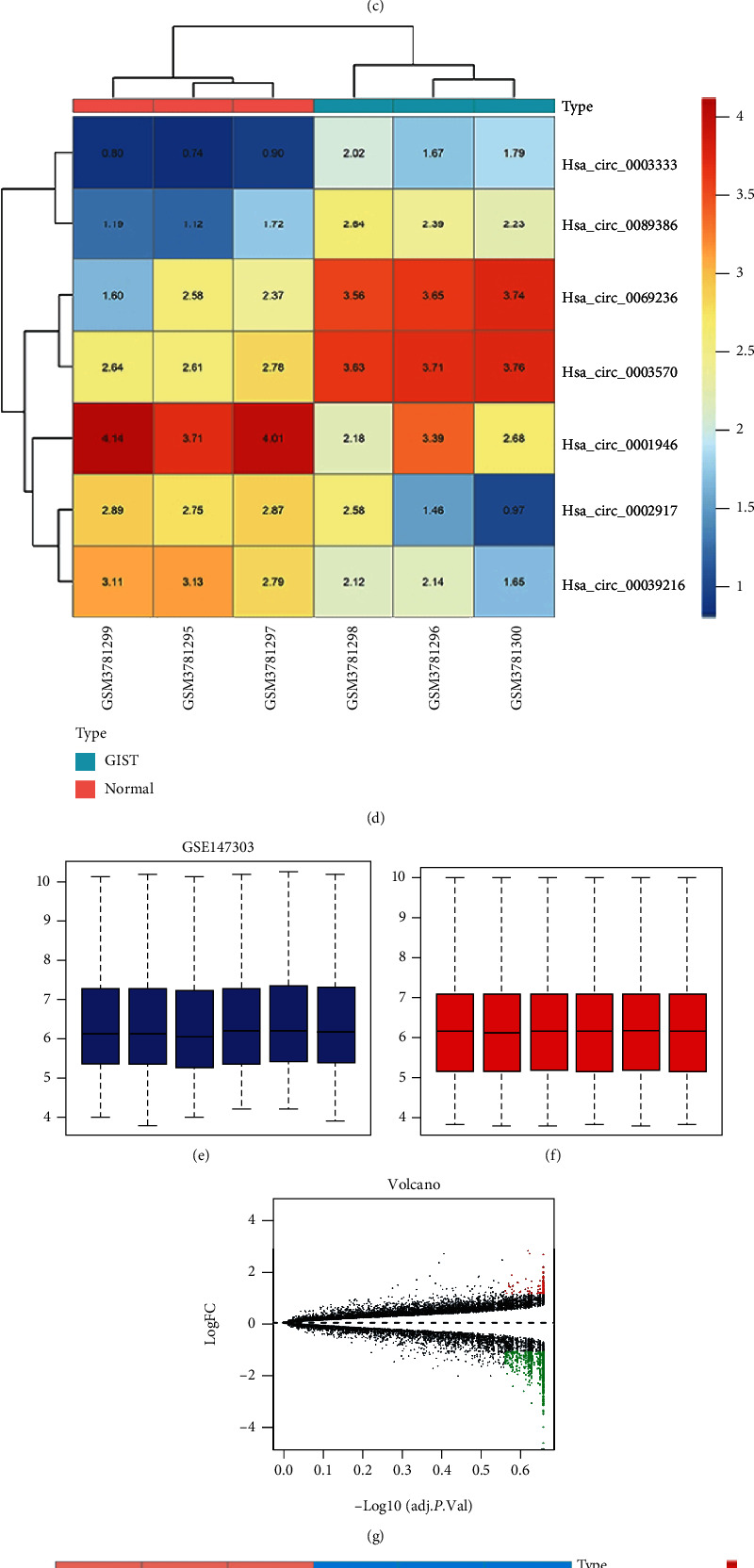
Differentially expressed circRNAs (DEcircRNAs). (a) Before the standardization of GSE 131481. (b) After the standardization of GSE131481. (c) Volcano plot of GSE131481. (d) Heatmap of GSE131481. (e) Before the standardization of GSE147303. (f) After the standardization of GSE147303. (g) Volcano plot of GSE147303. (h) Heatmap of GSE147303.

**Figure 2:**
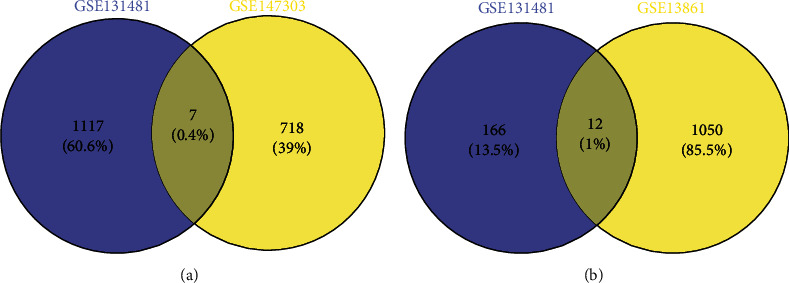
(a) The intersection of DEcircRNAs. (b) The intersection of DEGs.

The integrated analysis of GSE131481 and GSE147303 datasets from GEO database, respectively, identified 1124 and 725 DEcircRNAs. The selection criteria of ∣log2FC | >1 and *p* value < 0.05 were the differential expression circRNAs (DEcircRNAs) by R studio Limma packages; the GSE131481 included 717 upregulated and 407 downregulated circRNAs (Figures 1(a)–1(d)); the GSE147303 had 137 and 588 (Figures 1(e)–1(h)). Seven generating DEcircRNAs were found (Figure 2(a)), including hsa-circRNA-069236, hsa-circRNA-003333, hsa-circRNA-089386, hsa-circRNA-100709, hsa-circRNA-105055, hsa-circRNA-101273, and hsa-circRNA-101802; the outcomes with the 7 DEcircRNAs are shown in Table 1. Besides, we also used R studio Limma package analysis on GSE13861 and GSE131481 to obtain differentially expressed genes (DEGs). GSE13861 generated 1062 DEGs with ∣log2FC | >2 and adj*p*value < 0.05, 528 of which were upregulated and 534 of which were downregulated. Similarly, GSE131481 generated 178 differentially expressed DEGs with ∣log2FC | >1 and adjpvalue < 0.05, including 122 upregulated and 56 downregulated mRNAs. The DEGs obtained from the two datasets were intersected to get 12 DEGs (Figure 2(b)), which were PTPRH, PLN, ATP1A2, CLDN23, TSPAN2, CFD, ALDH1A1, MT1E, ADH1C, PHLDB2, SCNN1B, and KCNMA1.

### 3.2. GO and KEGG Analysis of DEGs

**Figure 3:**
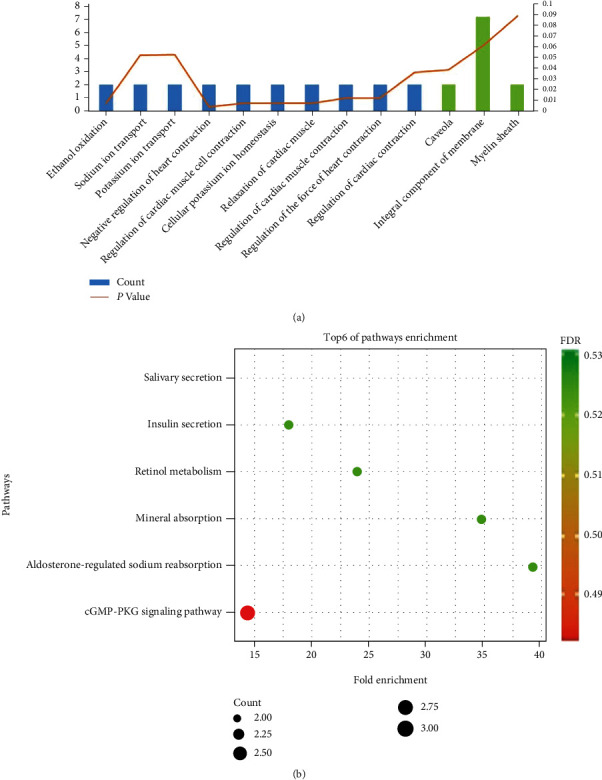
(a) GO analysis of DEGs in GIST. Thin blue bars represent biological processes (BP), and green bars represent cellular components (CC). The orange line represents *p* value. (b) KEGG analysis of DEGs in GIST.

In the GO result, the functions of DEGs include biological processes (BP) and cellular components (CC). GO enrichment demonstrated that DEGs were consisted of negative regulation of heart contraction, management in cardiac muscle cell contraction, ethanol oxidation, cellular potassium ion homeostasis, relaxation of cardiac muscle, regulation of myocardial contraction by regulating the release of chelated calcium ions, regulating myocardial contractility, and other components (Figure 3(a)). KEGG result determined that major DEGs were with cGMP-PKG signaling pathway (Figure 3(b)).

### 3.3. PPI Network and Hub Gene

**Figure 4:**
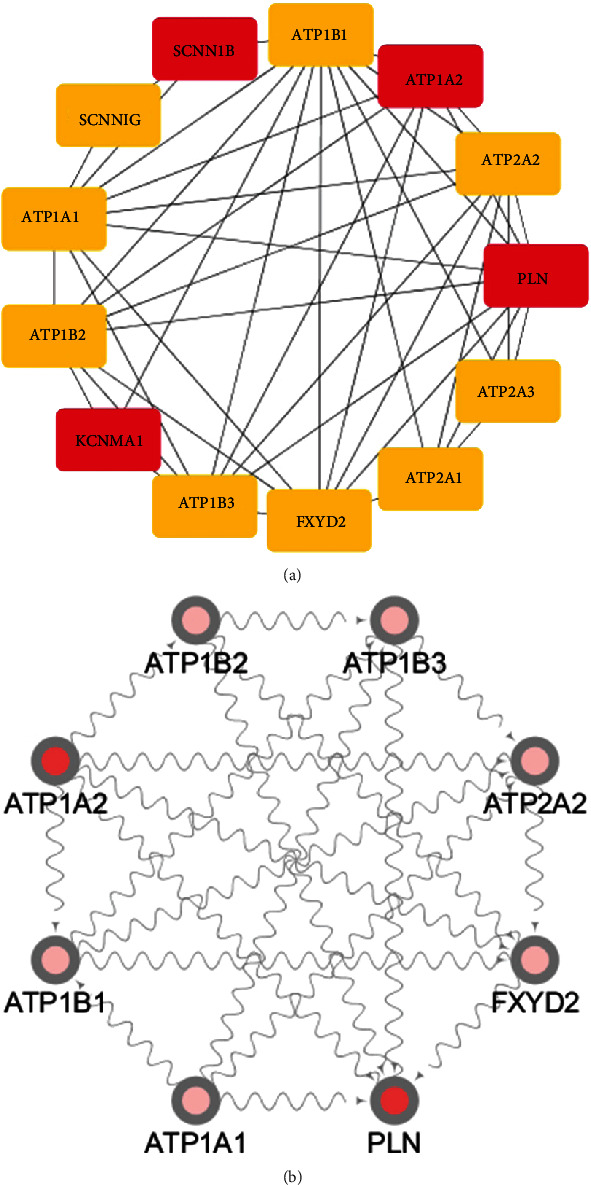
(a) The first four hub genes of 12 DEGs in cytoHubba. (b) Two hub genes in MCODE.

The 12 DEGs were putted into the STRING database to probe the interrelation between the all kinds of genes. PPI network was established using 12 DEGs. The PPI network involved 42 nodes and 97 edges. In cytoHubba, the first four hub genes were screened out according to the MCC algorithm, including ATP1A2, PLN, KCNMA1, and SCNN1B (Figure 4(a)). Two genes were screened out in MCODE, which were PLN and ATP1A2. According to the importance degree, a key module was selected from the PPI network by MCODE plug-in (Figure 4(b)). Furthermore, the results revealed that two of the four central DEGs (ATP1A2, PLN, KCNMA1, and SCNN1B) of the PPI network were created in this module, thus suggesting that PLN and ATP1A1 may have a significant important role in gastrointestinal stromal tumors.

### 3.4. Prediction of miRNA Binding Sites

**Figure 5:**
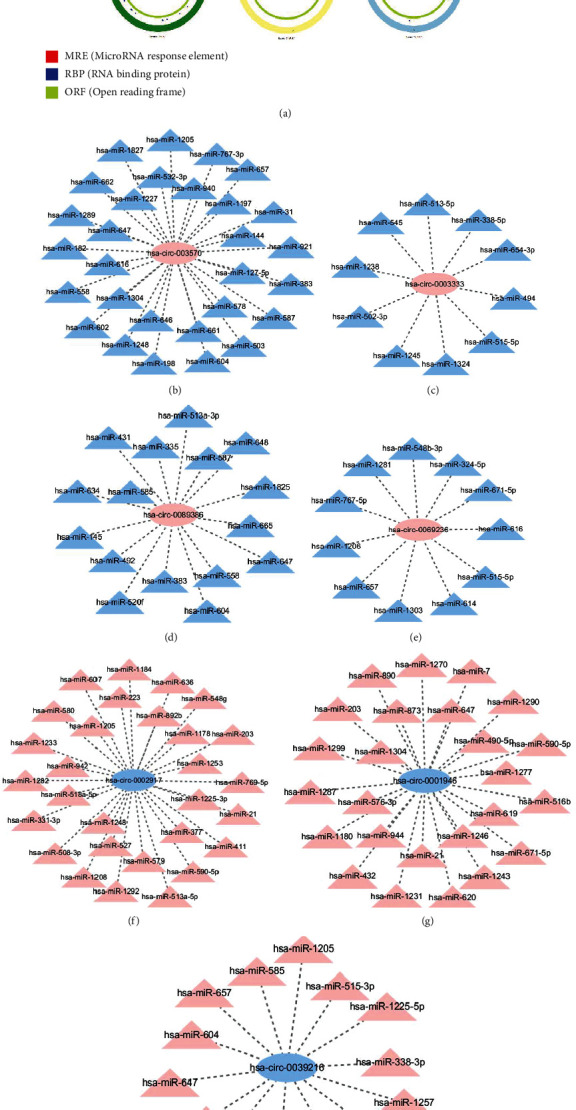
(a) The structure of DEcircRNAs. The red, blue, and green regions inside the circular RNA molecule, respectively, represent MRE (microRNA response element), RBP (RNA-binding protein), and ORF (open reading frame). (b–h) The circRNA-miRNA network diagrams. Pink represents upregulated DEcircRNAs, and blue represents downregulated DEcircRNAs.

**Figure 6:**
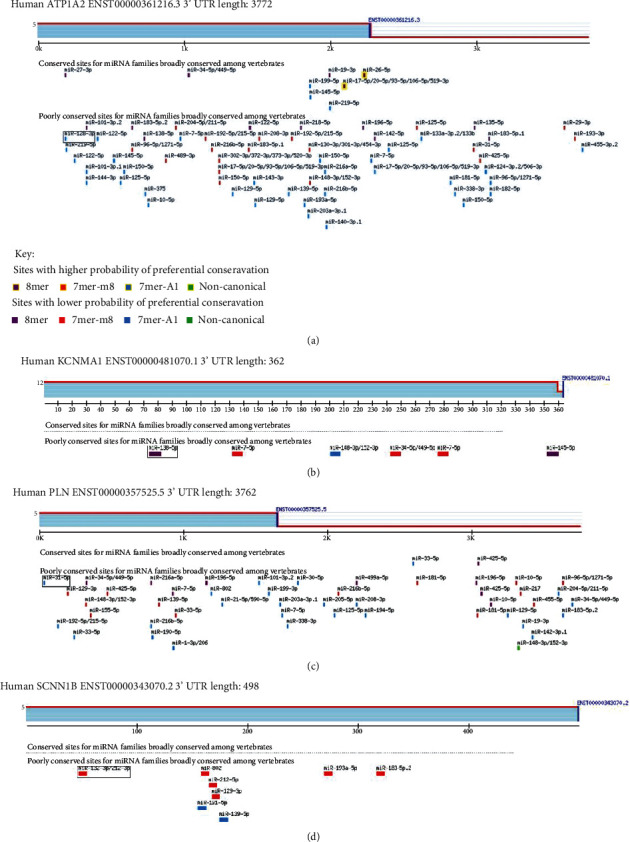
(a) The miRNA-binding of ATP1A2. (b) The miRNA-binding of KCNMA1. (c) The miRNA-binding of PLN. (d) The miRNA-binding of SCNN1B.

**Figure 7:**
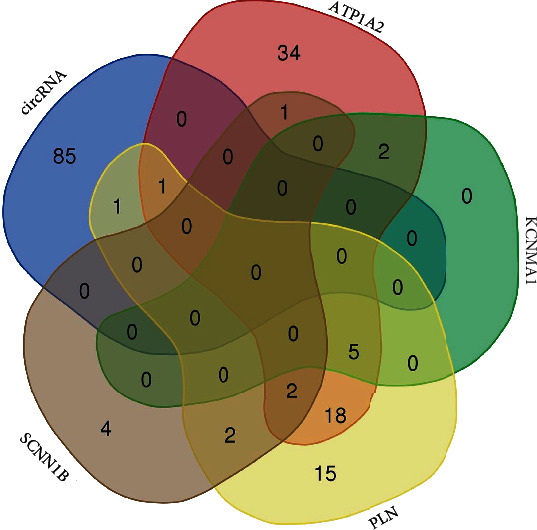
The intersection of miRNA. ATP1A2 had 63 targeted miRNA, PLN had 44 targeted miRNA, KCNMA1 had 7 targeted miRNA, and SCNN1B had 9 targeted miRNA. circRNA identified 87 miRNAs.

The structure of 7 DEcircRNAs was determined using the CSCD database, and these DEcircRNAs have MREs (Figure 5(a)). There were 87 miRNAs by Circular RNA Interactome. For better visualization, the circRNA-miRNA network diagram was conducted using Cytoscape software (Figures 5(b)–5(h)). The miRNA-binding of ATP1A2, PLN, KCNMA1, and SCNN1B was predicted by the TargetScan database (Figures 6(a)–6(d)). ATP1A2 has 63 targeted miRNA, PLN has 44 targeted miRNA, KCNMA1 had 7 targeted miRNA, and SCNN1B had 9 targeted miRNA. We intersected with miRNAs and then identified 2 DEmiRNAs in two databases (Circular RNA Interactome and TargetScan), namely, hsa-miR-338-3p and hsa-miR-590-5p (Figure 7).

### 3.5. A Potential circRNA-miRNA-mRNA Regulatory Network

**Figure 8:**
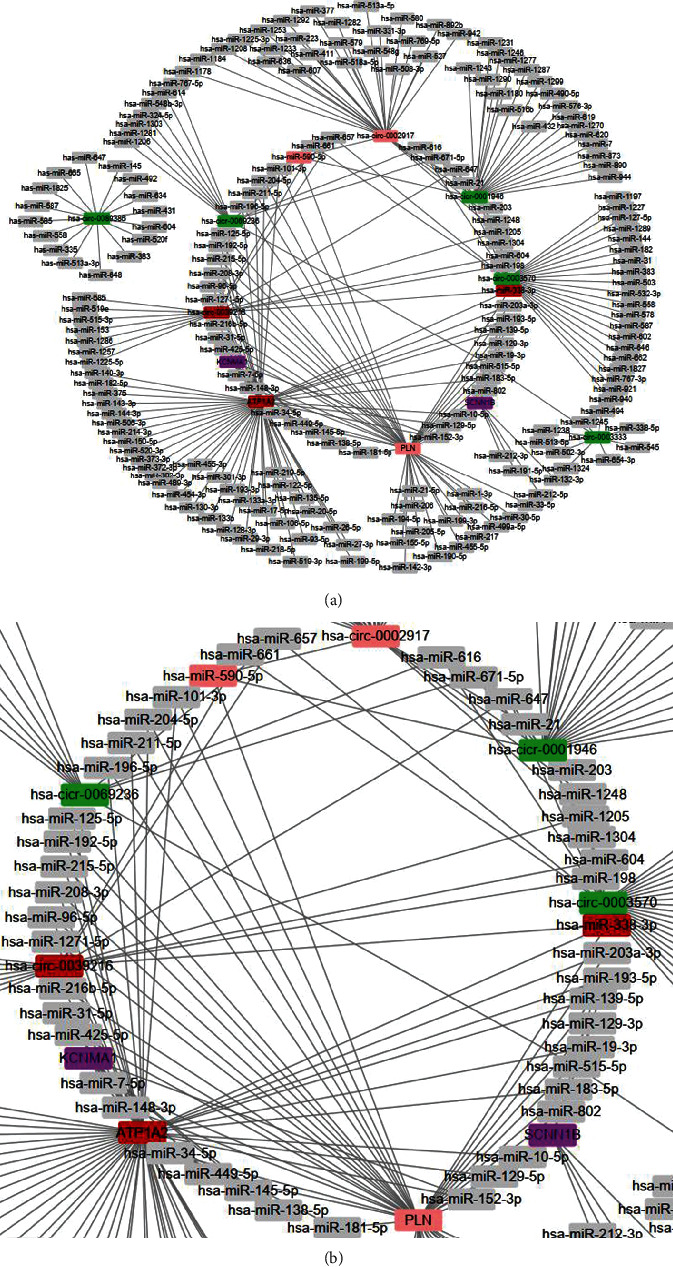
The circRNA-miRNA-mRNA regulatory network. (a) Overview of the entire network. (b) Specific relationship between circRNAs, miRNAs, and mRNAs in the network. Red represents hsa-circ-0039216/hsa-miR-338-3p/ATP1A2. Pink represents hsa-circ-0002917/hsa-miR-590/PLN. Purple represents DEGs. Green represents DEcircRNAs.

A growing number of researches have suggested that by competition for endogenous RNAs (ceRNAs), circRNAs can contend with miRNAs to affect the stabilizing of mark mRNAs or their transcription. In this study, 7 DEcircRNAs, 170 miRNAs, and 4 DEGs were obtained to compose the circRNA-miRNA-mRNA network (Figure 8). There are involved 87 interactions between DEcircRNAs and miRNAs. The genes of the 4 chosen mRNAs were predicted 85 mRNA-related miRNAs. The circRNA-miRNA-mRNA network was visible by Cytoscape software. Two circRNA/miRNA/mRNA axes were formed. Then, we got hsa-circ-0039216/hsa-miR-338-3p/ATP1A2 and hsa-circ-0002917/hsa-miR-590-5p/PLN axes, which may provide perspectives for the underlying mechanisms of GIST.

### 3.6. Validation of Hub Genes by qPCR

**Figure 9:**
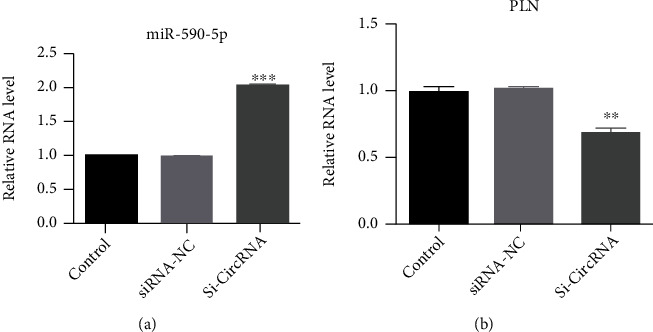
Validation of hub Genes by qRT-PCR. (a) The level of miR-590-5p expression. (b) The level of PLN expression. Black represents control group. Light grey represents transfection hsa-circ-0002917 siRNA negative control of gastrointestinal stromal tumor cells. Dark grey represents transfection hsa-circ-0002917 siRNA gastrointestinal stromal tumor cells. All data were expressed as the mean ± SD. ^∗^*P* < 0.05.

Our previous studies showed that circTBC1D4 was evidently upregulated, suggesting that circTBC1D4 was involved in the pathological process of GIST. So circTBC1D4 was chosen to study the physiological function of GIST by the qPCR method. After the low expression of circ TBC1D4 was constructed by siRNA, the miR-590 was significantly increased (Figure 9(a)). However, the PLN was significantly reduced (Figure 9(b)).

## 4. Discussion

GIST originates from the interstitial cells of Cajal [15]. Current epidemiology shows that the total incidence of GIST has been steadily increasing every year [16]. Recent studies have shown that common mutations including KIT, PDGFRA, and other DNA (e.g., BRAF and SDH) have become an indispensable part of GIST treatment and management [17]. It has been suggested that molecular biomarkers can provide guidance for the postoperative treatment of GIST and can improve the prognosis of GIST patients [18]. However, more researches were on the treatment of gastrointestinal stromal tumors. In this study, we identified DEcircRNAs and DEGs between GIST and adjacent normal gastric tissues and took the intersection. The circRNA targeting miRNAs intersected with the binding miRNAs of mRNAs, and two DEmiRNAs were obtained. The targeted mRNAs corresponding to the two DEmiRNAs were PLN and ATP1A2 that were exactly the same as the two mRNAs of the key modules selected in MCODE. Therefore, we assumed that hsa-circ-0039216/hsa-miR-338-3p/ATP1A2 and hsa-circ-0002917/hsa-miR-590-5p/PLN may have an important role in GIST.

circRNAs belong to competing endogenous RNAs (ceRNAs) [19] and as miRNA sponges to control gene expression by adsorbing microRNAs (miRNAs) as miRNA response elements (MRE) [12]. circRNAs, which are characterized by inherent stability, high conservatism, and generality, are an important biomarker for the screening, diagnosis, and prediction of digestive system tumors. Patel et al. [16] found that hsa-circ-0001013 was importantly increased in gastric cancer tissues relative to paired nontumor tissues, discovering as a new landmarker for gastric cancer diagnosis. Zhang et al. [20] demonstrated that circRNA-100269 targeting miR-100269 was decreased in gastric cancer and inhibited tumor cell. Furthermore, Zhang and colleagues [21] reported that circNrip1 promoted gastric cancer progression as a microRNA-149-5p sponge through the Akt1/mTOR pathway. More and more studies have confirmed the unusual expression of circRNAs in various tumors and the regulatory role of the circRNA-miRNA-mRNA network; nevertheless, there are less reports on circRNA-miRNA-mRNA networks in GIST. Contrasted to previous reports, we discovered that hsa-circ-0039216 and hsa-circ-0002917 may have a central role in regulating the progress of GIST. The mechanism underlying molecular targeting in GIST needs to be further elucidated. In this study, ATP1A2 and PLN were identified as the key genes related to GIST in the PPI network.

ATP1A2 is a catalytic part of active enzymes, which hydrolyzes ATP and promotes the exchange of sodium and potassium ions on the plasma membrane. This function produces electrochemical gradients of sodium and potassium that provide energy for the transport of various nutrients belonging to the ATPase family of transporters. In a dose-dependent manner, exogenous ATP depolarized the resting membrane and generated a tonic inward pacing current. An external sodium-free solution could inhibit the effect of ATP on pacing current. Clearance of exogenous Ca(2+) or thapsigargin, an inhibiting of endoplasmic reticulum Ca(2+) uptake, inhibits the effect of ATP on pacing current. The spontaneous [Ca(2+)] I oscillation can be enhanced by exogenous ATP. These results suggest that exogenous ATP regulates pacing cell activity by activating nonselective cationic channels through exogenous Ca(2+) influx and release of the endoplasmic reticulum [Ca(2+)]. Gastrointestinal stromal tumors arise from fusiform mesenchymal cells of Cajal stromal cells (ICC) or stem cell precursors of these cells [22]. Cajal interstitial cells exist in the stands and peripheral nerve to the muscularis propria gastrointestinal pacemaker cells. We hypothesized that ATP has an upregulating role in gastrointestinal stromal tumors [23].

Phospholamban (PLN), a member of the phospholamban family, regulates calcium reuptake during muscle relaxation. Phosphorylation of phospholamban (PLN) by Ca(2+)/calmodulin-stimulated protein kinase II (CaMKI) accelerates the sarcoplasmic reticulum (SR) Ca(2+)-ATPase (SERCA), which increases the rate of sarcoplasmic Ca(2+) clearance and intracellular calcium release. In most cancer cells, calcium reservoir-operated calcium ion influx mediates the majority of calcium ion influx and may be a factor in regulating intracellular calcium in Cajal and gastrointestinal stromal tumors. Therefore, blocking this mechanism may affect the progress of gastrointestinal stromal tumor [24]. Kurten et al. [25] found that the gene expression of fibronectin and smooth muscle actin was risen in eosinophilic esophagitis. The degree of ATP2A2 inhibition was determined by PLN oligomeric state, and phosphorylation of phosphatidylinositol could reduce the inhibition of ATP2A2. Li et al. [26] found that multiple expression of ATP2A2 was correlation with better prognostic in patients with diffuse astrocyte tumors. Wang et al. [27] found that phospholipid protein and inositol 1,4,5-triphosphate receptor 1 were upregulated and sarcoplasmic reticulum calcium transporter ATPase 2a was downregulated by sodium dextran sulfate-induced rat colitis model. We hypothesized that PLN has an upregulating role in GIST. Based on the GSE13861 and GSE131481 datasets, we found that ATP1A2 and PLN had significantly different expression patterns in GIST tissues. Therefore, it was necessary to further study the role of ATP1A2 and PLN in GIST to indicate the mechanism of ATP1A2 and PLN in regulating the physiological activity of GIST.

In recent years, studies found that miR-590-5p played an important role in digestive system diseases. Chen et al. [28] reported that miR-590-5p played an important effect in pancreatic adenocarcinoma cell lines. Zheng et al. [29] found that miR-590-5p could play a role in eliminating gastric cancer. Chen et al. [30] suggested that lncRNA FTX could promote colorectal cancer cell migration and invasion through miRNA-590-5p/RBPJ Axis. We found that circTBC1D4 affected the occurrence and development of GIST through the hsa-miR-590-5p/PLN axis. Through qRT-PCR experiments, the expression of miR-590-5p was high and the expression of PLN was low after TBC1D4 knockout.

In conclusion, this study revealed a potential circRNA-miRNA-mRNA network mediated by hsa-circ-0039216/hsa-circ-0002917 in gastrointestinal stromal tumors through total transcriptome analysis, cross-analysis, and correlation analysis. We identified ATP1A2 and PLN as potential inflammatory targets for the therapy of GIST. Our analysis and further experimental verification show that circTBC1D4 promotes the progression of gastrointestinal stromal tumor by regulating miR-590-5p/PLN axis in GIST, providing new insights for GIST. We will conduct further experiments to verify our conclusion.

## Figures and Tables

**Table 1:** The host gene symbol of DEcircRNAs.

circRNA ID	circBase ID	Gene symbol
hsa-circRNA-069236	hsa-circ-0069236	PROM1
hsa-circRNA-003333	hsa-circ-0003333	MCTP2
hsa-circRNA-089386	hsa-circ-0089386	VAV2
hsa-circRNA-100709	hsa-circ-0003570	FAM53B
hsa-circRNA-105055	hsa-circ-0001946	CDR1
hsa-circRNA-101273	hsa-circ-0002917	TBC1D4
hsa-circRNA-101802	hsa-circ-0039216	GPT2

## Data Availability

The datasets generated and/or analyzed during the current study are available in the NCBI GEO repository, GSE131481 (https://www.ncbi.nlm.nih.gov/geo/query/acc.cgi?acc=GSE131481), GSE147303 (https://www.ncbi.nlm.nih.gov/geo/query/acc.cgi?acc=GSE147303), and GSE13861 (https://www.ncbi.nlm.nih.gov/geo/query/acc.cgi?acc=GSE13861).
